# If You Go Down to the Woods Today: Infants’ Distress During a Teddy Bear's Picnic in Relation to Peer Relations and Later Emotional Problems

**DOI:** 10.1111/infa.12172

**Published:** 2016-11-24

**Authors:** Dale F. Hay, Stephanie H. M. van Goozen, Lisa Mundy, Rebecca Phillips, Siwan Roberts, Mirjam Meeuwsen, Ian Goodyer, Oliver Perra

**Affiliations:** ^1^ Cardiff University; ^2^ Melbourne Children's Hospital; ^3^ Exeter University; ^4^ Child and Adolescent Mental Health Services Betsi Cadwalladr Health Board; ^5^ University of Gottingen; ^6^ Cambridge University; ^7^ Queen's University Belfast

## Abstract

Infants’ emotional reactions to an unusual event were assessed at a simulated birthday party during which two costumed characters enacted a Teddy Bear's Picnic. Two hundred and fifty‐eight firstborn infants in a representative British community sample were observed at a mean age of 12.8 months in the presence of their parents and other participating families, in a laboratory sitting room decorated with balloons and banners. The picnic scenario was followed by free play with the other participating infants. At a mean of 36 months of age, mothers, fathers, and another informant who knew the child well completed the Child Behaviour Check List (CBCL). The majority of infants showed no vocal distress during the picnic scenario. A minority of infants expressed strong distress, which was correlated with elevated heart rate and production of cortisol. Infants who were not distressed were more likely to direct social behavior to their peers and especially likely to use physical force against peers. In comparison with strongly distressed and nondistressed infants, those who had shown mild distress during the picnic scenario were least likely to manifest later emotional problems. This pattern was particularly marked for boys. Taken together, the findings indicate that infants’ strong distress during naturalistic encounters that are meant to be entertaining can suppress sociability and might indicate risk for subsequent emotional problems.

Infants react to the unexpected in different ways. Fearfulness in response to novel environments predicts shyness with peers in later childhood (Fox, Henderson, Rubin, Calkins, & Schmidt, 2001) and anxiety in young adulthood (Frenkel et al., 2015). In contrast, infants who show fearless reactions to novel stimuli are more likely to have conduct problems in later childhood (e.g., Baker et al., 2013; Buss, Kiel, Morales, & Robinson, 2014; Dollar & Buss, 2014), especially if they also expressed positive affect (Buss et al., 2014). However, the evidence is mixed: Conduct‐disordered children who eventually developed callous‐unemotional traits actually showed elevated stress in response to novelty when they were infants (Mills‐Koonce et al., 2015).

The novel settings used in previous research have been specially designed to elicit fearful reactions. In contrast, we presented 12‐month‐old infants and their families with a social situation that could possibly evoke pleasure, interest, or distress: a simulated birthday party with costumed characters, a fairy‐tale princess, and a teddy bear (Figure 1). The Teddy Bear's Picnic paradigm simulates experiences in everyday life, when children meet costumed characters at a party, fundraising event, or amusement park. The design of the study did not equate to an experimental stress‐reactivity paradigm. Rather, infants were presented with a set of experimental procedures across an afternoon (see also Luby et al., 2003 for a similar design), with individual testing preceding the simulated birthday party. Because the Teddy Bear's Picnic might evoke pleasure in some infants and distress in others, positive affect (smiling or laughing) was recorded as well as distressed vocalizations (fussing or crying).

**Figure 1:**
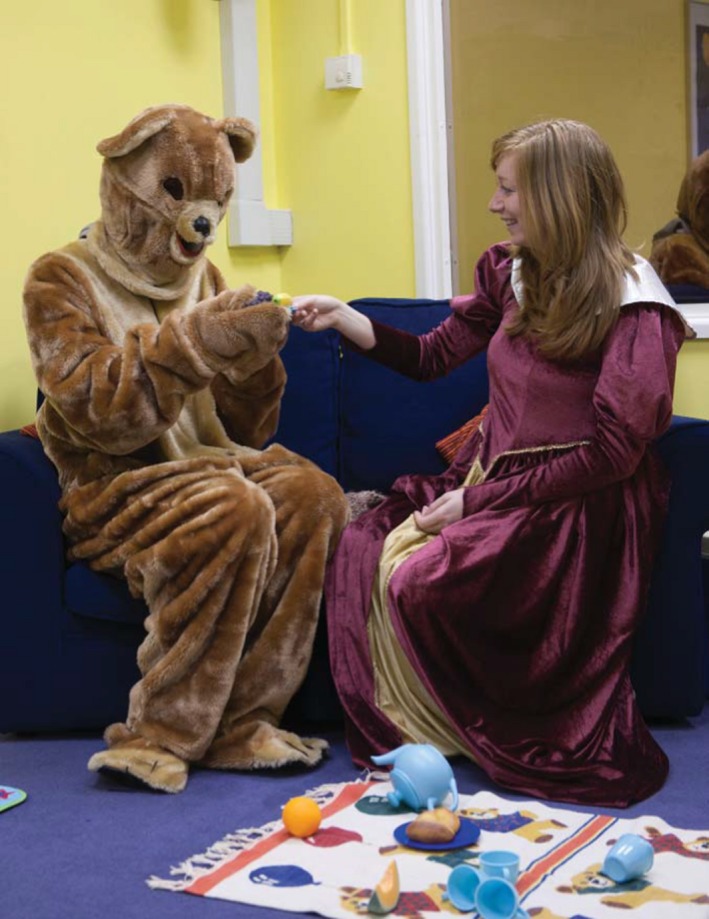
Birthday lady and teddy bear.

In examining infants’ reactions to the Teddy Bear's Picnic scenario, we have considered distress to be a multidimensional phenomenon. Infants’ observable reactions to novelty have physiological correlates (e.g., Buss, Davidson, Kalin, & Goldsmith, 2004). Therefore, our first aim was to examine whether infants’ overt distress in response to the picnic scenario was indeed associated with elevated heart rate and/or elevated cortisol levels. Evidence for associations between the infants’ overt behavior and either of these physiological responses would be of interest, given that not all studies have found links between overt display of emotion and underlying physiology (Barrett, 2015). Because heart rate and cortisol production depend on different biological systems, there was no expectation that the two physiological measures would themselves be correlated.

The second aim of our study was to detect immediate effects of infants’ reactions to the picnic scenario on their social behavior with new acquaintances. If, as the literature suggests, fearful reactions to novel stimuli signal risk for later shyness while fearless responses predict aggression, are infants’ reactions to unusual events such as the picnic scenario already influencing their social behavior, especially their reactions to unfamiliar children? Two‐year‐olds who showed fear in response to novelty were also more anxious when they later met up with other toddlers (Rubin, Burgess, & Hastings, 2002). However, infants’ emotional reactions to novelty may affect their social behavior at an even earlier age, when their interest in peers is first emerging (Ross, Vickar, & Perlman, 2010). If links between responses to novelty and peer relations are already evident in infancy, that fact would have consequences for all infants who spend time with other infants, either in childcare settings or their wider community networks.

Because prior research has shown that infants’ fearless responses to novelty predict later aggression (Buss et al., 2014), we tested the specific hypothesis that infants’ distressed reactions would be inversely correlated with their use of physical force against the peers (other infants in the sample who attended the birthday party). Infants’ use of force against other infants emerges by 10 months (Eckerman, Whatley, & Kutz, 1975) and is correlated with known risk factors for aggression (Hay et al., 2011). However, in very early childhood, the use of force is sometimes positively correlated with more positive forms of sociability (e.g., Green, 1933), and so it is important to consider whether infants’ distress might suppress all forms of peer‐directed behavior, not just the use of force. Distressed infants might also be less likely to engage in positive interaction with their peers.

It is also possible that infants’ positive affect during the picnic might facilitate the use of force as well as more positive behavior. Children's exuberant responses to novel challenges are sometimes associated with elevated levels of aggression (e.g., Dollar & Stifter, 2012). Thus, we examined associations between the infants’ distress and positive affect during the picnic scenario and their subsequent forceful and positive behavior with peers immediately thereafter.

The third aim of the study was to examine developmental continuities from infants’ distress in reaction to the Teddy Bear's Picnic scenario to their later risk for psychological problems as toddlers. In view of the fact that infants’ reactions to novel situations predict later developmental outcomes (e.g., Frenkel et al., 2015), it seemed likely that infants who were distressed by the Teddy Bear's Picnic paradigm would show a greater number of emotional problems as toddlers. It is important to note, however, that previous work suggests that the extent of continuity from early reactions to novel situations to later outcomes might be moderated by the child's gender (e.g., Henderson, Fox, & Rubin, 2001). Therefore, we have tested whether the extent of continuity from distressed reactions in infancy to emotional problems in early childhood was different for girls and boys.

Furthermore, when testing for a link between early distress in response to novel situations and later emotional problems, it is important to recall that emotional and behavioral problems are often highly correlated in early childhood (e.g., Eisenberg et al., 2001). We must ask, does distress in response to novelty in infancy predict later emotional problems, even when controlling for co‐occurring aggressive conduct problems? If so, we will have identified an early marker for a subset of emotional problems that are not necessarily bound up with frustration and aggression.

Finally, in testing the hypotheses, it is sensible to move beyond the dichotomy of distress vs. no distress to novelty and differentiate mild and strong distressed reactions to the novel situation. It is not unreasonable to show some discomfort or wariness when a strange bear walks into a room and so infants who show mild signs of distress may be at less risk for later emotional problems than those who react more strongly.

In summary, we examined infants’ vocal distress and physiological reactions to a novel situation that might provoke distress or amusement. We examined the associations between infants’ reactions to the novel situation and their subsequent interaction with peers as well as their later risk for emotional problems. We tested all hypotheses in the context of a longitudinal study of firstborn British infants.

## Method

### Design

Families expecting their first child were recruited in the third trimester of pregnancy when all mothers and 86% of their partners were assessed. Infants were then observed in an alternating sequence of home visits and laboratory visits at a mean of 6, 12, 21, and 33 months of age. Up to three informants per family, including those unable to visit the laboratory, reported on the child's emotional and behavioral problems at a mean of 36 months.

### Participants

The study was approved by the U.K. National Health Service (NHS) Multi‐Centre Research Ethics Committee. *N *=* *332 primiparous women were recruited from antenatal services clinics in two NHS Trusts. Potential participants provided contact addresses and postcodes. Both those who chose to participate and those who chose not to participate in the study represented the entire range of socioeconomic categories associated with U.K. postal codes. Demographic characteristics of the sample are presented in Table 1. The final sample was nationally representative on key socioeconomic variables, not differing significantly from first‐time parents in the most recent U.K. national cohort study (K. Kiernan, personal communication, April 2009).

**Table 1:** Demographic Characteristics at Wave 1 for Full Sample and Subsample of Infants Assessed at 12 and 36 Months

Variable	Total sample	12 months	36 months
Mother's age at birth (Mean)	28.15	28.79	28.82
Stable partnerships (%)	90.4	90.3	92.5
Marital status (% legally married)	50.3	55.0	54.7
Ethnicity (% British or Irish)	92.7	92.7	92.3
Social class (% middle class)	50.9	56.2	54.7
Mother's education (% ≥ basic qualifications)	80.0	81.8	81.9

In the subsample of families assessed in the Teddy Bear’s Picnic paradigm, the mean socioeconomic adversity factor score (−.11) did not differ significantly from the original mean of the full sample (0). The mean socioeconomic adversity factor score (−.12) for the families who provided questionnaire data at a mean of 36 months did not differ significantly from the mean of those participating in the Teddy Bear’s Picnic paradigm.

**Figure 2:**
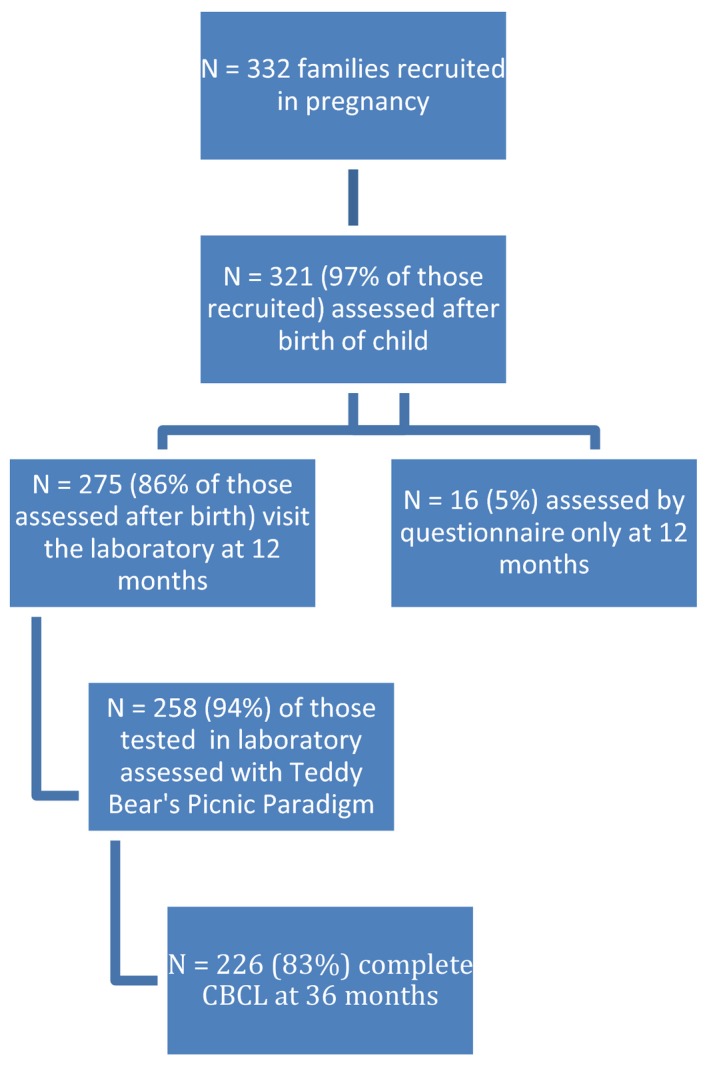
Participation in the Teddy Bear’s Picnic paradigm.

Of the 332 families who were recruited in pregnancy, 321 (97%) continued to participate in the study after the child's birth. Of those, 291 (91%) were assessed at a mean of 12.8 (*SD* 1.1) months, with 275 (45% girls) visiting the laboratory and 16 providing questionnaire data only (Figure 2). Nine families could not be traced within the time window (11–14 months), and eight others were not assessed due to work commitments, poor health, or acute family problems. Another 12 families booked laboratory visits, but canceled and were unable to reschedule within the assessment time window. In four cases, infants took part in individual testing but could not be assessed during the birthday party paradigm, due to other families having canceled.

Emotional reactions to the Teddy Bear's Picnic Paradigm were scored for 258 (94%) of the 271 infants who participated in the 12‐month birthday party. Six did not receive the full procedure, due to procedural refinements. Video problems occurred in seven other cases. The families who were assessed at 12 months were not significantly different from the original sample on socioeconomic variables.

At a mean of 36 months of age, 240 mothers, 179 fathers, and 184 family members or friends completed questionnaires for 254 children. Of the 271 families who had been able to attend the birthday party at 12 months, 226 (83%) provided questionnaire data at 36 months.

### Procedure and measures

#### Adverse family circumstances

Mothers and 80% of biological fathers reported on their family circumstances via interview and questionnaires during the pregnancy. A general index of the child's exposure to factors known to be associated with sociodemographic adversity was created using a polychoric principal component analysis on the following variables: (1) the mother not having achieved basic educational attainments, that is, no qualifications or fewer than five General Certificate of Secondary Education (GCSE) examinations passed or equivalent; (2) the mother being 19 years of age or under at the child's birth; (3) the parents not being legally married; (4) the parents not being in a stable couple relationship; and (5) the mother's occupation being classified as working class according to the Standard Occupational Classification 2000 (SOC2000; Elias, McKnight, & Kinshott, 1999). All these items contributed to a single component that explained approximately 77% of the shared variance in these risk indicators. Summary scores on that component were used as a general measure of socioeconomic adversity (see comparison of original sample and this subsample in Table 1).

#### Laboratory visit at 12 months

At a mean of 12.8 months postpartum (*SD* 1.1), families were invited to visit the School of Psychology. Three families were scheduled for each testing session, which began at 2 PM and was approximately 1½ h in duration.

Infants were first assessed in separate testing rooms in the presence of their caregivers (90% mothers) for 25 min, using a battery of cognitive and social communication tasks. The infants were then brought together with their parents and other families to a large, comfortably furnished sitting room, decorated with banners and balloons for a birthday party. Depending on cancellations and rescheduling of participants, two to four infants were present at each party. If one family had canceled, the infants from the two attending families were observed together. If two families canceled, the infant from the third family was assessed individually and invited to attend another scheduled party, which on a few occasions resulted in a group of four infants with their family members. Primary caregivers often invited their partners or extended family members to attend the party, resulting in the complex social situation that had been envisioned.

The theme of the party was a Teddy Bear's Picnic. Two costumed characters (the “Birthday Lady” and the “Teddy Bear”) entered the room and encouraged the infants to share plastic picnic food and dance with the teddy bear (Figure 1). The birthday lady was a researcher dressed as a fairy‐tale princess, wearing a medieval costume and carrying a picnic basket. She greeted the infants and began setting out the contents of her picnic basket on a small woven mat decorated with teddy bears. The basket contained a tea set, plates, and play food items. The birthday lady encouraged all infants to sit around the picnic mat to play with the plastic food and all parents to return to one of the sofas in the room. A researcher dressed in a bear costume then entered the sitting room, pausing in the doorway until each infant had seen the bear.

The bear's designated persona was that of an active toddler whose playful and social behavior was supervised by the birthday lady. The bear did not speak to the infants, and its nonverbal actions were interpreted by the birthday lady. The birthday lady invited the teddy bear to sit down near the picnic blanket, and the bear offered each infant a piece of plastic birthday cake. The birthday lady and bear then performed a dance while singing “Round and Round the Garden” before offering each infant, with the help of their parents, the opportunity to dance with and tickle the bear at the end of the song. Once each infant had danced with the birthday lady and bear, the bear offered to shake hands with each infant in turn and then left the play room. The birthday lady instructed the families to let the infants play in any way they would like for 20 min and then she also left the room.

Throughout the procedure, parents were instructed to act as they normally would when their infant came into contact with a costumed character, for example, at a community fundraising event or during a visit to a theme park.

#### Infants’ distress and positive affect

Infants’ vocal distress in response to the Teddy Bear’s Picnic was recorded using the Distress Observation System (DOS), which had been adapted from a coding system used to record vocal distress in older toddlers (Demetriou & Hay, 2004). Observers (trained postgraduate and undergraduate students) recorded infants’ mild (whimpering, whining, fussing) and strong (crying, weeping, screaming) distress and positive affect (smiling, laughing) during each of the following epochs of the birthday party scenario: (1) the birthday lady's invitation to the picnic and distribution of play food; (2) the teddy bear's entrance to the playroom; (3) the teddy bear's offering of the picnic food; (4) the “Round and Round the Garden” dance; and (5) the teddy bear's offering to shake hands to say good‐bye. The following summary measures of infants’ behavior during Epochs 2–5 (after the bear entered the room) were computed: (1) any *strong vocal distress*,* κ *= .77; (2) the *level of vocal distress* shown on a three‐point scale (none, mild, and strong), Intraclass Correlation Coefficient (ICC) = .92; and (3) any sign of *positive affect*,* κ *= .79. The measure of strong vocal distress permits a categorical comparison of infants who cried with all other members of the sample. The scale score of level of distress allowed for a more nuanced comparison of those who showed strong, mild, and no distress in response to the picnic scenario. Distress and positive affect during the first epoch where the birthday lady set out the picnic is reported separately for purposes of comparison. For ethical reasons, if an infant had already begun to cry as soon as the bear entered the room, that infant was judged to have met the criteria for “strong vocal distress” and was not asked to participate in the remaining epochs of the picnic scenario prior to the bear leaving the room (i.e., dancing with or being touched by the bear), although the paradigm would continue for any other nondistressed infants in the room. All infants took part in the assessment of peer interaction following the picnic scenario, regardless of their levels of distress during the picnic.

#### Heart rate

Heart rate (HR) data were collected using the ActiGraph ActiTrainer (Actigraph LLC, Fort Walton Beach, FL, USA) worn in conjunction with a Polar Wearlink T31 device connected to a chest strap. The electrode areas of the Polar strap were moistened with water before use. The Polar strap was attached around the infants’ sternum and secured so that it was a snug fit, with the Polar logo on the connector positioned in a central and upright position. The ActiGraph ActiTrainer has dimensions of 8.6 cm by 3.3 cm by 1.5 cm and weighs approximately 1.8 ounces and was attached to the infants’ upper left leg with a Velcro strap. Once the Polar strap was attached securely, it created a unique coded communication link with the ActiGraph ActiTrainer. This unique link prevented data from other Polar straps corrupting the HR data.

The ActiGraph ActiTrainer collected and stored the HR data transmitted from the Polar strap, while at the same time recording accelerations. The sampling rate was 30 Hz. Heart rate data were stored as the average beats‐per‐minute per epoch. Epochs were defined as 15‐sec blocks in line with recommendations to allow for detection of normal human motion, and 15‐sec epochs have previously been used in studies of preschool‐aged children (e.g., Pate, Almeida, McIver, Pfeiffer, & Dowda, 2006). The HR data were downloaded via the integrated USB plug, stored in ASCII format and subsequently converted into a Microsoft Excel file with the Actilife Software. The data were cleaned, and average heartbeats per minute were calculated for (1) a baseline period of 3 min, (2) a 3‐min period during individual testing when the infant explored a novel toy, and (3) for the duration of the Teddy Bear's Picnic scenario. The baseline period was collected at the start of the individual testing session of the infant before any other tasks were completed. The durations of the baseline period, the novel toy period, and the bear visit were verified by matching up timing sheets and video recordings. For the purpose of these analyses, we focus on the HR data collected concurrently with the Teddy Bear's Picnic scenario.

Of the 271 infants who participated in the birthday party, 255 (94%) provided HR data. Three participated before the birthday party procedure had been finalized; eight wore the first version of the infant actigraph to be developed by the company, which measured activity but not HR; two refused to wear the actigraph; and in seven cases, there were technical faults or procedural errors. A logarithmic transformation was applied to the HR data to improve normality of the distributions.

Because the actigraph simultaneously measured HR and motor activity, it was possible to control for motor artifact when analyzing changing patterns of HR over the afternoon of testing. The ActiGraph ActiTrainer contained an activity monitor with a built‐in single axis accelerometer, which was designed to measure and record time varying accelerations ranging in magnitude from approximately 0.05–2 Gs. The output from the accelerometer was digitized by a 12‐bit analog‐to‐digital converter (ADC) at a rate of thirty times per second, that is, 30 Hertz (Hz). When the signal had been digitized, it passed through a digital filter which band limits the accelerometer to the frequency range of 0.25–2.5 Hz. These limits were chosen carefully to allow detection of normal human motion but reject motion from other sources. Each sample was then summed over a 15‐sec epoch (the same timing of epochs was used for the measurement of motor activity and HR). Activity during the Teddy Bear's Picnic procedure was averaged over epochs and used as a covariate in analyses of HR.

#### Cortisol

To avoid confounding infants’ response to the birthday party paradigm with the expected diurnal rhythms in cortisol production, all infants were tested between 2 and 4 PM in the afternoon. Cortisol samples were collected at three points in the afternoon: at entrance to the laboratory; at the end of the 25 min of individual testing; and at the end of the birthday party, 20 min after the Teddy Bear’s Picnic procedure had ended. Cortisol was collected and each infant's actigraph removed prior to the Lucky Dip procedure that ended the party. The timings of the cortisol samples allow us to infer that the first assessment reflects the infant's earlier baseline state, the second assessment reflects reactions to individual testing in the laboratory, and the third reflects reaction to the Teddy Bear's Picnic procedure that occurred 20 min earlier (see Waters et al., 2013 for further information on the trends in cortisol production over time).

Each saliva sample collection took no longer than 2 min. Sorbettes and cryovials (Salimetrics, State College, PA, USA) were used for collecting the saliva from the infant's mouth. Oral stimulants to increase saliva flow were not used to avoid contamination (Schwartz, Granger, Susman, Gunnar, & Laird, 1998). In light of evidence that milk in saliva can interfere with the cortisol assay (Magano, Diamond, & Gardner, 1989), the parents were informed that their children could not be fed during the experimental procedure. Due to the ages of the children and the likelihood of teething, caution was taken to report any indications of blood in the mouth or on the sorbettes, which could result in elevated cortisol levels.

Samples were immediately stored and frozen at −20°C until they were shipped in dry ice for analysis. All samples were assayed with Elisa cortisol assays. Thawed saliva samples were spun at 1,500 rpm for 15 min at 4°C and assayed in duplicate. A standard curve was generated for every plate of samples assayed. The average intra‐ and interassay coefficients of variation were 4.33 and 9.25%, respectively.

Of 275 infants who visited the laboratory, 257 (93%) provided at least one usable saliva sample, with *N *=* *204 cases (74%) providing a usable sample at each data collection point. The subsample with complete data was not significantly different from the overall sample on demographic characteristics. Exploratory data analyses identified four outliers, which were removed. A logarithmic transformation was applied to the cortisol data to improve the normality of the distributions.

#### Peer interaction

The Teddy Bear's Picnic scenario was followed by 20 min of free play with parents and peers. Parents were instructed to chat together as they would if they met other parents for the first time at an actual birthday party and otherwise to respond to their infants as they naturally would during such a party. The procedure was filmed using two wall‐mounted cameras. Infants’ peer‐directed behavior during the 20 min of free play was coded using the Peer Interaction Coding System, which had previously been used with 12‐month‐olds (PICS; Caplan, Vespo, Pedersen, & Hay, 1991). Postgraduate and undergraduate observers viewed video records, using event sampling to identify episodes in which infants directed social behavior to each other, as defined by a standard set of behavioral categories of predefined actions. Episodes of peer‐directed behaviors comprising alternating moves made by each infant began when one peer‐directed action shown by one infant was followed by a peer's socially directed behavior and ended with the last move preceding at least 30 sec in which no peer‐directed moves were recorded. The observers who coded peer interaction were not the same individuals who had coded the infants’ vocal distress.

To test the hypothesis that distress in response to the Teddy Bear’s Picnic might be inversely correlated with infants’ subsequent peer‐directed behavior and especially their use of physical force, three summary measures of infants’ peer‐directed behavior were constructed: (1) *offering* objects to a peer; (2) *tugging* on toys held by a peer; and (3) use of *bodily force* against a peer's body (pushing, pulling, or striking out at the peer). Offering is a positive communicative gesture that does not entail the use of physical force; tugging on toys and use of bodily force both involve the use of physical force with the peers. Observers recorded whether each peer‐directed move definitely or possibly contained any of these three behaviors, yielding a summary score for each type of force. Transcripts made by independent observers (a team of trained undergraduate students) for 61 (22%) cases revealed acceptable levels of observer agreement, ICC = .88 for offering/giving objects to peers, ICC = .80 for tugging on peers’ toys, and ICC = .79 for the use of bodily force. To improve the normality of the distributions, each measure was subjected to a square root transformation for further analyses.

Preliminary analyses on the square root transformed data showed that the infants’ peer‐directed behavior was not significantly associated with group size. Mixed‐model analyses of each category of peer‐directed behavior were used to check for dependencies in the data, that is, whether the infants’ scores were affected by being assessed at particular parties in the presence of particular peers, by testing for the random effect of group membership. The analyses showed that the infants’ frequency of tugging on toys was significantly affected by being paired with particular peers (when one infant tugged on another's toy, the possessor was likely to tug it back), but offers and use of bodily force showed no such effect of group membership. Because the bodily force and offering scores showed no dependencies in the data, they were analyzed further as measures of the infants’ individual tendencies to direct physical force vs. a more positive social behavior to their peers. Both measures were dichotomized.

#### Lucky Dip task

Following the free play session, the final cortisol sample was taken and the actigraphs were removed. The infants and their parents were then asked to participate in a “Lucky Dip” in which each infant could delve into a box filled with plastic balls to find a birthday present (an age‐appropriate book). Parents were given £20 vouchers for participating in this wave of the study.

#### Emotional and behavioral problems in toddlerhood

Of the 258 families assessed in the Teddy Bear's Picnic paradigm at 12 months, 215 (83%) completed questionnaires at 36 months, using the 1½‐ to 5‐year‐old version of the Child Behavior Check List (CBCL; Achenbach & Rescorla, 2000). The CBCL aggressive problems scale yields an age‐appropriate continuous measure of young children's aggressiveness and associated conduct problems (mean *α *= .90 across the three informants). The two CBCL syndrome scales for *emotionally reactive* symptoms and *anxious/depressed* symptoms, which were highly intercorrelated, were combined to form a composite measure of emotional problems (mean *α *= .73 across the three informants). Mothers’ ratings on the aggressive problems scale were significantly correlated with fathers’ ratings, *r* (168) = .46, *p *<* *.001, and with ratings provided by the other family member or friend, *r* (172) = .48, *p *<* *.001. Fathers’ ratings were significantly associated with those made by the third informant, *r* (150) = .39, *p *<* *.001. Similarly, for the composite emotional problems scale, mothers’ ratings were significantly correlated with fathers’ ratings, *r* (168) = .25, *p *<* *.001, and with the third informant's ratings, *r* (172) = .28, *p *<* *.001. Fathers’ ratings were significantly correlated with the third informant's ratings, *r* (150) = .23, *p *<* *.005. Summary factor scores for the aggressive and emotional problems scales were derived by a measurement model using Mplus 7 to integrate the information provided by all available informants.

### Data analysis

All study variables were examined for psychometric properties and scores transformed where appropriate, as described above. Descriptive statistics for untransformed scores of all measures of the children's behavior are presented in Table 2. All correlational and longitudinal analyses were conducted using Full Information Maximum Likelihood (FIML) methods, which allow the estimation of the likelihood function from complete and incomplete observations. All families that contributed some data at any point after the child's birth (*N *=* *321) were included in these analyses despite incomplete observations. The use of FIML methods rests on the assumption of data missing at random (MAR), or alternatively the assumption that missing information is related to other observed variables but not related to the unobserved value of the outcome of interest. This assumption was deemed reasonable. To facilitate estimation of missing values, we also included auxiliary variables in these analyses, as noted in relevant tables below. The auxiliary variables were not included as covariates in the models, but rather used to explain the mechanism of missingness and thus help estimate missing values of variables. Covariates included factors that might influence social behavior and risk for psychological problems, that is, socioeconomic adversity and gender, as well as controls for baseline levels of the physiological levels and methodological variance, for example, physical activity levels which might influence actigraph readings.

**Table 2:** Descriptive Statistics for Raw Score Measures of Children's Behavior

Behavioral reactions to the Teddy Bear's Picnic	
Highest level of distress	64.7% no distress; 15.9% mild; 19.4% strong
Positive affect	34.1% positive affect
Physiological reactions, Mean (*SD*)	
Mean heart rate at baseline (bpm)	132.81 (21.04)
Mean heart rate during picnic	141.16 (15.21)
Cortisol level at baseline (ng/ml)	2.03 (2.59)
Cortisol level after picnic	2.48 (3.33)
Peer‐directed behavior at 12 months, Mean (*SD*)	
Offers to peer	3.34 (5.78)
Tugging on peers’ toys	2.32 (4.08)
Bodily force against peer	1.16 (2.82)
Psychological problems at 36 months, Mean (*SD*)	
Mean CBCL emotional problems	3.62 (2.45)
Mean CBCL aggressive problems	8.20 (5.17)

These FIML methods were used to examine the three main research questions in the study. Firstly, we asked whether infants’ overt vocal distress was a marker of a more multifaceted distress response, that is, whether it was correlated with HR and cortisol responses to the novel situation. Thus, the physiological measures were used to validate the infants’ overt vocal distress as a marker of distressed responses to the Teddy Bear's Picnic, which was then carried forward as the key independent variable in the subsequent analyses.

The second set of analyses tested the hypothesis that infants’ distressed behavior in reaction to the novel situation would immediately suppress their social behavior with peers, particularly their use of force against peers. These analyses were conducted on dichotomized measures, thus yielding clear information about the odds of peer‐directed behavior following strong distress, while controlling for other relevant covariates.

The final set of analyses tested the longitudinal hypothesis that infants’ level of distress (none, mild distress, or strong distress) predicted later emotional problems at 36 months of age. The analysis controlled for co‐occurring aggressive conduct problems and other risk factors and tested whether the link between distress in infancy and later emotional problems was moderated by gender.

## Results

### Infants’ behavioral reactions to the Teddy Bear’s Picnic

#### Vocal distress

During the initial phase of the scenario, when the birthday lady invited the infants to help unpack the picnic basket, only 11 of the 258 infants (4%) showed mild distress and 12 (5%) cried. In contrast, after the teddy bear entered the room, 91 infants (35%) showed either mild or strong vocal distress, with 50 (19%) becoming strongly distressed at some point during the bear's visit. No gender differences were observed, and infants’ distress was unrelated to the size of the peer group. Because of the low frequency of distress while the birthday lady was setting out the picnic and the fact that observers’ notes indicated that any distress that did occur was often caused by infants’ motor problems or their peers’ actions, the first epoch of the picnic was not analyzed further. Subsequent analyses focus on infants’ behavior and physiological reactions after the teddy bear entered the playroom.

#### Positive affect

Eighty‐eight infants (34%) smiled or laughed at least once after the bear entered the room. Girls and boys did not differ. The expression of positive affect was inversely related to strong distress, *κ *= −.23, *p *<* *.001. Only five children (2%) who had shown strong distress also expressed positive affect.

### Associations between overt distress and physiological measures of arousal

The first major research question concerned the examination of overt vocal distress as a measure of a multifaceted response to the novel situation in relation to physiological measures.

#### Heart rate

The HR scores were log‐transformed, and implausible scores were removed for four cases. FIML regression analyses (Table 3) indicated that infants’ level of vocal distress was significantly correlated with mean HR (log‐transformed) during the Teddy Bear's Picnic, even when controlling for baseline HR, gender, exposure to adversity, and actigraph‐measured physical activity during the picnic procedure, *β *= .23, *z *=* *4.68*, p *<* *.001. Inclusion of overt distress in the model contributed to an increase in explained variance (*R* ^2^) from .35 to .40 and a significant improvement of model fit, *LR* test *χ* ^2^(1) = 20.27, *p *<* *.001. In contrast, infants’ positive affect was unrelated to HR. The FIML model also indicated a negative association between HR during the Teddy Bear's Picnic and being male; the mean HR for males was reduced by a unit of 0.13 *SD* (Table 3).

**Table 3:** Standardized coefficients and 95% CI of Full Information Maximum Likelihood Regression on HR During the Teddy Bear's Picnic, *N *=* *321. The Log of HR During Exploration of a Novel Toy was used as an Auxiliary Variable to Estimate Missing Scores

Outcome: (log10)HR @ picnic	*β*	*SE*	*z*	95% CI
(log10)HR @ baseline	0.49	0.05	10.48***	0.40 to 0.58
(log10)Activity @ picnic	0.21	0.05	4.18***	0.11 to 0.31
Male	−0.13	0.05	−2.51*	−0.23 to −0.03
Adversity	0.004	0.05	0.08	−0.10 to 0.11
Vocal distress score	0.23	0.05	4.68***	0.14 to 0.33
Intercept	25.97	3.61	7.19***	18.87 to 33.01

Model Log likelihood = 464.1; LR test (model vs. saturated): *χ* ^2^(2) = 9.29, *p *=* *.002.

*p <* *.05; **p <* *.01; ***p <* *.001.

#### Cortisol levels

The cortisol readings were log‐transformed and two outliers were removed. The FIML model included gender, family adversity (power transformed to improve normality of residuals), and baseline cortisol as covariates. Cortisol levels during the individual testing session were used as an auxiliary variable to help estimate missing scores. After controlling for covariates, there was a significant association between vocal distress and infants’ levels of cortisol during the Teddy Bear's Picnic (Table 4). The final model explained approximately 18% of variance. Taken together, the analyses of both HR and cortisol further validated the observational measure of vocal distress as a fearful response to the picnic scenario. Subsequent analyses focus on the short‐term and longer‐term correlates of the infants’ vocal distress.

**Table 4:** Standardized Coefficients and 95% CI of Full Information Maximum Likelihood Regression on Cortisol Levels During the Teddy Bear's Picnic, *N *=* *321. The Log of Cortisol Levels During the Individual Testing Session was Included as an Auxiliary Variable to Help Estimate Missing Values

Outcome: (log)Cortisol @ Picnic	*β*	*SE*	*z*	95% CI
(log)Cortisol @ baseline	0.38	0.06	6.35***	0.26 to 0.50
Male	−0.02	0.06	−0.26	−0.14 to 0.10
(power)Adversity	0.05	0.06	0.82	−0.07 to 0.17
Vocal distress score	0.15	0.06	2.43*	0.03 to 0.28
Intercept	0.36	0.16	2.28*	0.05 to 0.66

Model Log likelihood = −1521.9; LR test (model vs. saturated): *χ* ^2^(1) = 20.80, *p *<* *.001.

*p <* *.05; **p <* *.01; ***p <* *.001.

### Immediate effects of distress during the picnic on infants’ peer‐directed behavior

In the second set of analyses, we tested the hypothesis that infants’ distress during the Teddy Bear's Picnic might immediately suppress interaction with unfamiliar peers.

#### Descriptive analyses

Initial analyses showed that 50% of the infants offered toys to their peers; 43% tugged on peers’ toys; and 20% used bodily force against their peers. As noted earlier, tugging on peers’ toys was not examined further, due to dependencies in the data. Bodily force was *positively* correlated with infants’ tendencies to offer toys to their peers, *κ *= .13, *p *<* *.01.

Girls were significantly more likely than boys to offer toys to their peers, with 57% of girls vs. 44% of boys making offers, *χ* ^2^(1) = 4.54, *p *<* *.05, *OR* 1.30 (95% CI: 1.02–1.64). Girls and boys did not differ in the likelihood of tugging on peers’ toys or using bodily force against their peers.

Positive affect during the Teddy Bear's Picnic was unrelated to infants’ behavior with peers. However, strong distress was associated with less peer interaction. Only 34% of the strongly distressed infants (*N *=* *50) offered objects to peers, as opposed to 52% of the other infants, *χ* ^2^(1) = 5.18, *p *<* *.025, *OR* 1.53 (95% CI: 1.02–2.30).

Logistic regression analysis revealed that, after controlling for family adversity, as well as for the significant association with the infant's gender, infants who had not shown strong distress during the picnic were twice as likely to offer objects to their peers, *OR* = 2.18 (95% CI: 1.14–4.19), Wald statistic = 5.49, *p *=* *.02, Nagelkerke *R* ^2^ = .05. Family adversity did not significantly affect the likelihood of offers. However, gender remained a significant predictor of infants’ likelihood of offering toys to their peers.

Strong distress during the Teddy Bear's Picnic was associated with less bodily force: only four (8%) of the 50 strongly distressed infants, as opposed to 23% of the others, used bodily force, *χ* ^2^(1) = 5.42, *p *<* *.05. Logistic regression analysis controlling for family adversity revealed that the infants who had not been strongly distressed during the picnic were over three times as likely to use bodily force against their peers, *OR* = 3.37 (95% CI: 1.15–9.85), Wald statistic = 4.92, *p *=* *.03, Nagelkerke *R* ^2^ = .04. Family adversity did not significantly affect the likelihood of using bodily force against peers.

### Prediction of CBCL scores at 3 years

#### Descriptive analyses

The CBCL aggressive problems factor score and the composite emotional problems factor score were positively correlated, *r* (254) = .63, *p *<* *.001. The positive correlation was shown in the reports of all three informants, the magnitude of the correlation coefficients ranging from *r* (182) = .55, *p *<* *.001 for the third informants to *r* (176) = .67, *p *<* *.001 for fathers. The test of the hypothesis that infants’ distress would predict later emotional problems was conducted while controlling for co‐occurring aggressive conduct problems.

After preliminary regression diagnostics, the CBCL emotional problems factor score was log‐transformed. FIML regression analyses controlling for gender and family adversity (Table 5) indicated that mild levels of vocal distress during the Teddy Bear's Picnic at 12 months were associated with lower levels of emotional problems at 36 months *β *= −.19, *z *=* *−2.98, *p *=* *.003, and this effect was not moderated by gender. The model explained approximately 5% of variance. However, when CBCL aggression scores were taken into account, gender moderated the association between vocal distress in infancy and subsequent emotional problems. Strong vocal distress at 12 months in boys as compared to girls was associated with higher levels of emotional problems at 36 months, *β = *.16, *z = *−2.03, *p *=* *.042. This pattern of findings indicates the presence of suppression effects.

**Table 5:** Standardized Coefficients and 95% CI of Full Information Maximum Likelihood Regression on Child Behaviour Check List Emotional Problems at 36 Months, *N *=* *321

Outcome: (log)Emotional problems	*β*	*SE*	*z*	95% CI
Mild vocal distress 12 months	−0.20	0.07	−2.98**	−0.33 to −0.07
Strong vocal distress 12 months	0.04	0.07	0.62	−0.09 to 0.18
Male	0.01	0.06	0.12	−0.11 to 0.13
Adversity	0.08	0.07	1.23	−0.05 to 0.22
Intercept	2.00	0.14	14.64***	1.73 to 2.26

Outcome: (log)Emotional problems	*β*	*SE*	*z*	95% CI
Mild vocal distress 12 months	−0.08	0.08	−1.00	−0.24 to 0.08
Strong vocal distress 12 months	−0.06	0.08	−0.69	−0.21 to 0.10
Aggressive problems 36 months	0.74	0.12	5.82***	0.49 to 0.99
Male	−0.07	0.06	−1.10	−0.19 to 0.05
Adversity	−0.03	0.05	−0.50	−0.13 to 0.08
Aggressive problems × Male	−0.12	0.12	−1.03	−0.35 to 0.11
Male × Mild voc. distress	−0.06	0.08	−0.76	−0.22 to 0.10
Male × Strong voc. distress	0.20	0.09	2.36*	0.03 to 0.38
Aggressive problems × Mild voc. distress	0.01	0.09	0.09	−0.17 to 0.19
Aggressive problems × Strong voc. distress	−0.09	0.09	−1.00	−0.26 to 0.08
Aggressive Problems × Mild voc. distress × Male	0.03	0.08	0.32	−0.13 to 0.19
Aggressive problems × Strong voc. distress × Male	0.16	0.08	2.00*	0.003 to 0.31
Intercept	2.04	0.13	15.89***	1.79 to 2.29

In the first model, co‐occurring aggressive problems are not included. In the second model, co‐occurring aggressive problems at 36 months were controlled for.

Model Log likelihood (first model) = −986.2.

Model Log likelihood (second model) = −994.0.

*p <* *.05; **p <* *.01; ***p <* *.001.

**Figure 3:**
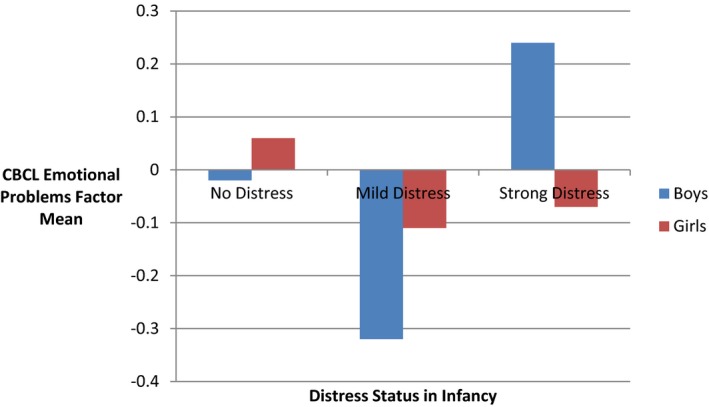
Level of distress during the Teddy Bear's Picnic and Child Behaviour Check List (CBCL) emotional problems at 36 months. Scores are adjusted for co‐occurring CBCL aggression scale.

Further analyses showed that the suppression effect was bound up with a three‐way interaction among gender, level of aggressiveness, and the level of vocal distress in infancy (Figure 3). When co‐occurring aggressive conduct problems were controlled for, model comparisons suggested an increase in fit when a three‐way interaction between level of vocal distress, gender, and aggressive problem scores was included. Boys who displayed strong vocal distress at 12 months manifested higher levels of emotional problems at 36 months when they also displayed co‐occurring aggressive problems. The final model explained approximately 44% of variance (Table 5).

## Discussion

Our study using a naturalistic birthday party paradigm extends previous longitudinal research that identified links between infants’ reactions to fear‐provoking novel situations (Fox et al., 2001; Rubin et al., 2002). Those infants who were most distressed by the picnic scenario also showed elevated cortisol and heart rate, which corroborated the stressful nature of the experience for some infants. In everyday life, it is not uncommon for infants to encounter costumed characters and other surprising events; our findings suggest that while some enjoy the experience, others become strongly distressed.

Those infants who were most distressed were subsequently less likely to offer toys to their peers and highly unlikely to strike out at their peers. Earlier evidence for links between early fearfulness and later social reticence (Fox et al., 2001; Rubin et al., 2002) could be explained by intervening variables, such as time spent in childcare or parents’ encouragement of peer interaction. However, our findings demonstrated that infants’ distress in response to the picnic paradigm is *immediately* followed by lower levels of peer‐directed behavior. To the extent that infants’ early experiences with peers foster their social cognitive skills (Ross et al., 2010), infants who are distressed by unusual situations may be disadvantaged in this regard.

In particular, distressed infants were less likely to use physical force against peers, a finding in line with prior evidence for a link between fearlessness in infancy and aggressive conduct problems (Baker et al., 2013; Buss et al., 2014; Dollar & Buss, 2014). Infants’ use of bodily force is indeed associated with known risk factors for aggression, especially mothers’ own history of conduct problems (Hay et al., 2011), and significantly associated with the infants’ general anger and use of force, as reported by family members (Hay et al., 2014). However, caution is needed before drawing the conclusion that the nondistressed infants were more aggressive. Bodily force was positively correlated with more common social overtures such as offering toys to other people, which were also suppressed by the infant's distress. It is safer to say that distress suppressed sociability.

The infants’ distress during the Teddy Bear's Picnic predicted their later risk for emotional problems. Those infants who showed mild distress when a large teddy bear walked into the room were least likely to show emotional problems as toddlers. Their wary reactions could be interpreted as a sign of developmentally appropriate appraisal and subsequent coping with a challenging social situation. In contrast, infants who were strongly distressed by the picnic paradigm were more likely to manifest emotional problems as toddlers. However, this form of continuity over time was influenced by the child's gender and co‐occurring levels of aggressiveness. The links between the nature of the distress reaction in infancy and the level of emotional problems in childhood were particularly marked for boys. This finding supports earlier research that continuities from infants’ distress reactions to later developmental outcomes are moderated by gender (Henderson et al., 2001). This pattern is obscured if co‐occurring aggression problems are not taken into account.

These findings provide a reminder that although behavioral and emotional problems are sometimes seen as conceptual opposites, being outwardly vs. internally directed, they are actually strongly related. The fact that distress was negatively related to infants’ use of force against their peers suggests that the aggressive problems shown by 3 years of age may emerge from earlier emotional vulnerability, but that possibility needs further test in this and other samples. Claims that fearless children are at risk for aggressiveness (e.g., Baker et al., 2013; Buss et al., 2014), whereas children who are distressed by novel events are at risk for social reticence and anxiety (Fox et al., 2001; Frenkel et al., 2015) may represent a false dichotomy.

Our study has several limitations. Because the participants had been invited to a birthday party, at least two families were present, and in many cases, the infants’ primary caregivers invited their partners or other extended family members to come along to the party. While this led to an ecologically valid paradigm, the number of people in the room limited possible camera angles, and therefore, fine‐grained coding of infants’ facial expressions was not feasible. In addition, because the infants were freely moving through the party wearing actigraphs, the equipment we used permitted us to record HR but not vagal tone or skin conductance, as investigated in other work on infants’ emotional responses to novelty (e.g., Baker et al., 2013; Rubin, Hastings, Stewart, Henderson, & Chen, 1997).

However, despite these limitations, it is clear that the Teddy Bear's Picnic paradigm evoked coherent individual differences at both a behavioral and physiological level that were linked to the infants’ immediate social engagement with peers and predicted later profiles of emotional problems. The findings have practical implications. As infants and their parents go about the world, they encounter situations that are intended to provide amusement but, for some infants, may be quite frightening. By 12 months, many infants routinely spend time with peers, and their behavior in those settings has implications for their later development. Those infants who are either entirely sanguine or highly distressed in the face of unusual events may already be on a developmental pathway toward psychological problems.
